# Supplementary material for: Resolving the full spectrum of human genome variation using Linked-Reads
Source: Genome Res. 2019 Apr;29(4):635–45. doi: 10.1101/gr.234443.118 (PMC6442396; doi:10.1101/gr.234443.118)
Supplement: Supplemental Material [file supp_gr.234443.118_Supplemental_longranger-2.2.zip › longranger-2.2.tar.bz2!/lr2.2-public/lib/stan/cmdstan/stan/doc/api/html/unit__e__metric_8hpp_source.html]

Stan: src/stan/mcmc/hmc/hamiltonians/unit\_e\_metric.hpp Source File


|  |  |
| --- | --- |
|  | Stan  2.5.0  probability, sampling & optimization |


- Main Page
- Related Pages
- Namespaces
- Classes
- Files

- File List
- File Members

All Classes Namespaces Files Functions Variables Typedefs Enumerations Enumerator Friends Macros Pages

- src
- stan
- mcmc
- hmc
- hamiltonians

unit\_e\_metric.hpp

Go to the documentation of this file.

1 #ifndef STAN\_\_MCMC\_\_UNIT\_\_E\_\_METRIC\_\_BETA

2 #define STAN\_\_MCMC\_\_UNIT\_\_E\_\_METRIC\_\_BETA

3

4 #include <boost/random/variate\_generator.hpp>

5 #include <boost/random/normal\_distribution.hpp>

6

7 #include <stan/mcmc/hmc/hamiltonians/base\_hamiltonian.hpp>

8 #include <stan/mcmc/hmc/hamiltonians/unit\_e\_point.hpp>

9

10 namespace stan {

11

12  namespace mcmc {

13

14  // Euclidean manifold with unit metric

15  template <typename M, typename BaseRNG>

16  class unit\_e\_metric: public base\_hamiltonian<M, unit\_e\_point, BaseRNG> {

17

18  public:

19

20  unit\_e\_metric(M& m, std::ostream\* e):

21  base\_hamiltonian<M, unit\_e\_point, BaseRNG>(m, e) {};

22  ~unit\_e\_metric() {};

23

24  double T(unit\_e\_point& z) {

25  return 0.5 \* z.p.squaredNorm();

26  }

27

28  double tau(unit\_e\_point& z) { return T(z); }

29  double phi(unit\_e\_point& z) { return this->V(z); }

30

31  const Eigen::VectorXd dtau\_dq(unit\_e\_point& z) {

32  return Eigen::VectorXd::Zero(this->model\_.num\_params\_r());

33  }

34

35  const Eigen::VectorXd dtau\_dp(unit\_e\_point& z) {

36  return z.p;

37  }

38

39  const Eigen::VectorXd dphi\_dq(unit\_e\_point& z) {

40  return z.g;

41  }

42

43  void sample\_p(unit\_e\_point& z, BaseRNG& rng) {

44

45  boost::variate\_generator<BaseRNG&, boost::normal\_distribution<> >

46  rand\_unit\_gaus(rng, boost::normal\_distribution<>());

47

48  for (int i = 0; i < z.p.size(); ++i)

49  z.p(i) = rand\_unit\_gaus();

50

51  }

52

53  };

54

55  } // mcmc

56

57 } // stan

58

59

60 #endif

stan::mcmc::base\_hamiltonian< M, unit\_e\_point, BaseRNG >::model\_

M & model\_

**Definition:** base\_hamiltonian.hpp:59

stan::mcmc::unit\_e\_metric::dtau\_dq

const Eigen::VectorXd dtau\_dq(unit\_e\_point &z)

**Definition:** unit\_e\_metric.hpp:31

base\_hamiltonian.hpp

stan::mcmc::base\_hamiltonian< M, unit\_e\_point, BaseRNG >::V

double V(unit\_e\_point &z)

**Definition:** base\_hamiltonian.hpp:25

stan::mcmc::unit\_e\_point

**Definition:** unit\_e\_point.hpp:12

stan::mcmc::unit\_e\_metric::phi

double phi(unit\_e\_point &z)

**Definition:** unit\_e\_metric.hpp:29

stan::mcmc::unit\_e\_metric::~unit\_e\_metric

~unit\_e\_metric()

**Definition:** unit\_e\_metric.hpp:22

stan::mcmc::ps\_point::p

Eigen::VectorXd p

**Definition:** ps\_point.hpp:48

stan::mcmc::unit\_e\_metric::dtau\_dp

const Eigen::VectorXd dtau\_dp(unit\_e\_point &z)

**Definition:** unit\_e\_metric.hpp:35

stan::mcmc::ps\_point::g

Eigen::VectorXd g

**Definition:** ps\_point.hpp:51

stan::mcmc::unit\_e\_metric::dphi\_dq

const Eigen::VectorXd dphi\_dq(unit\_e\_point &z)

**Definition:** unit\_e\_metric.hpp:39

stan::mcmc::unit\_e\_metric::tau

double tau(unit\_e\_point &z)

**Definition:** unit\_e\_metric.hpp:28

stan::mcmc::unit\_e\_metric::T

double T(unit\_e\_point &z)

**Definition:** unit\_e\_metric.hpp:24

stan::mcmc::base\_hamiltonian

**Definition:** base\_hamiltonian.hpp:17

stan::mcmc::unit\_e\_metric::sample\_p

void sample\_p(unit\_e\_point &z, BaseRNG &rng)

**Definition:** unit\_e\_metric.hpp:43

stan::math::e

double e()

Return the base of the natural logarithm.

**Definition:** constants.hpp:86

stan::mcmc::unit\_e\_metric::unit\_e\_metric

unit\_e\_metric(M &m, std::ostream \*e)

**Definition:** unit\_e\_metric.hpp:20

stan::mcmc::unit\_e\_metric

**Definition:** unit\_e\_metric.hpp:16

unit\_e\_point.hpp


---

[ Stan Home Page ]

*© 2011–2014,
Stan Development Team.*
